# Supplementary figures and images for: INO80 regulates chromatin accessibility to facilitate suppression of sex-linked gene expression during mouse spermatogenesis
Source: PLoS Genet. 2024 Oct 15;20(10):e1011431. doi: 10.1371/journal.pgen.1011431 (PMC11508167; doi:10.1371/journal.pgen.1011431)

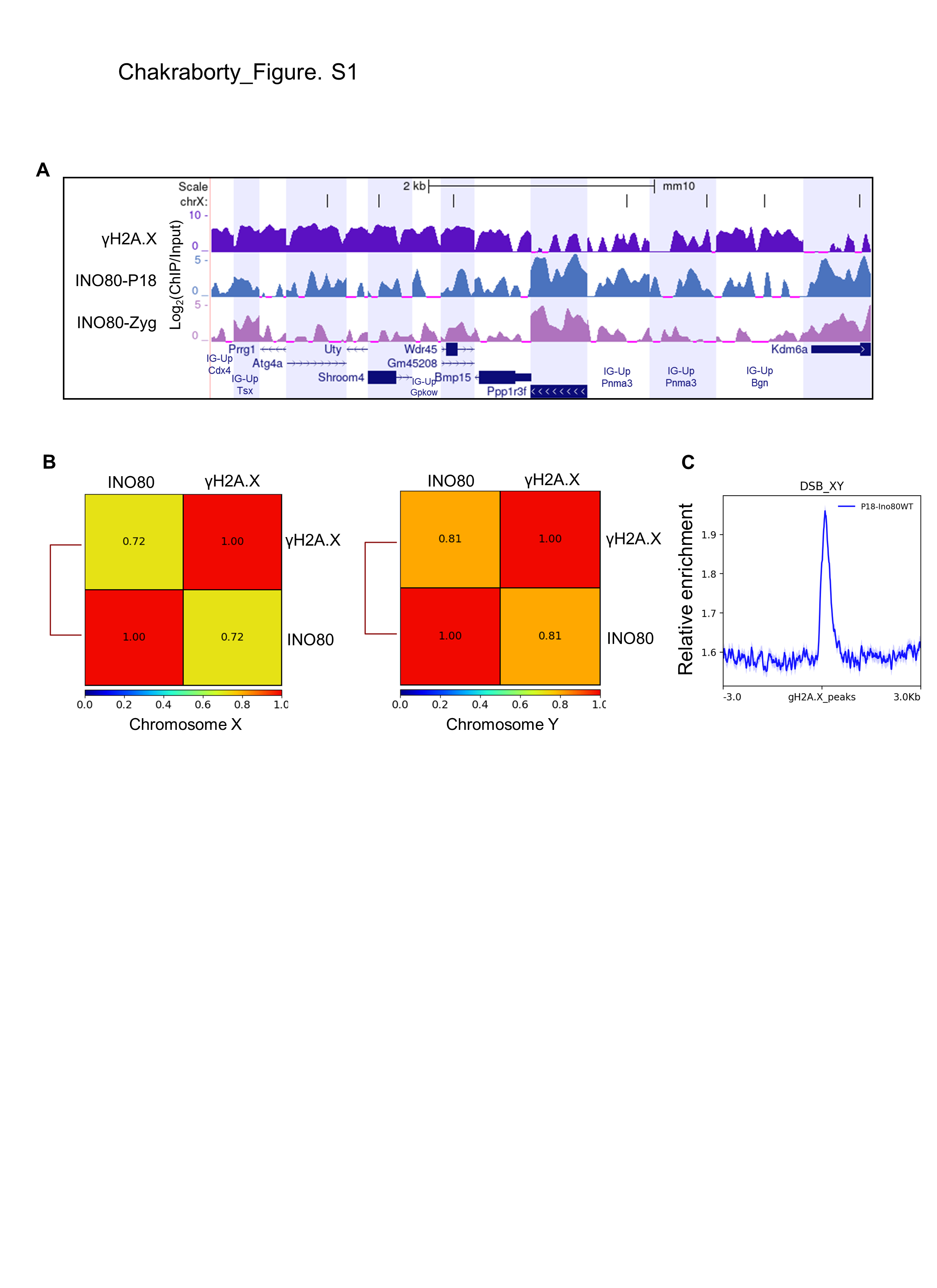

Supplement: S1 Fig — (A) Genomic tracks illustrating INO80 binding at the DSB sites marked by γH2A.X [34] in P18 [32] and zygotene [33] spermatocytes. Each separate genomic location is denoted by alternating background coloring. IG-Up; Intergenic-Upstream. (B) Correlation analysis of INO80 and γH2A.X binding at the X and Y chromosomes. The numbers in the box represent Pearson’s correlation coefficient calculated from high confidence reads (mapping quality >30) mapped to either chromosome X or Y. (C) Metaplot showing INO80 occupancy in Ino80WT spermatocytes at the sex chromosome DSB sites marked by γH2A.X. (TIF) [file pgen.1011431.s001.TIF]

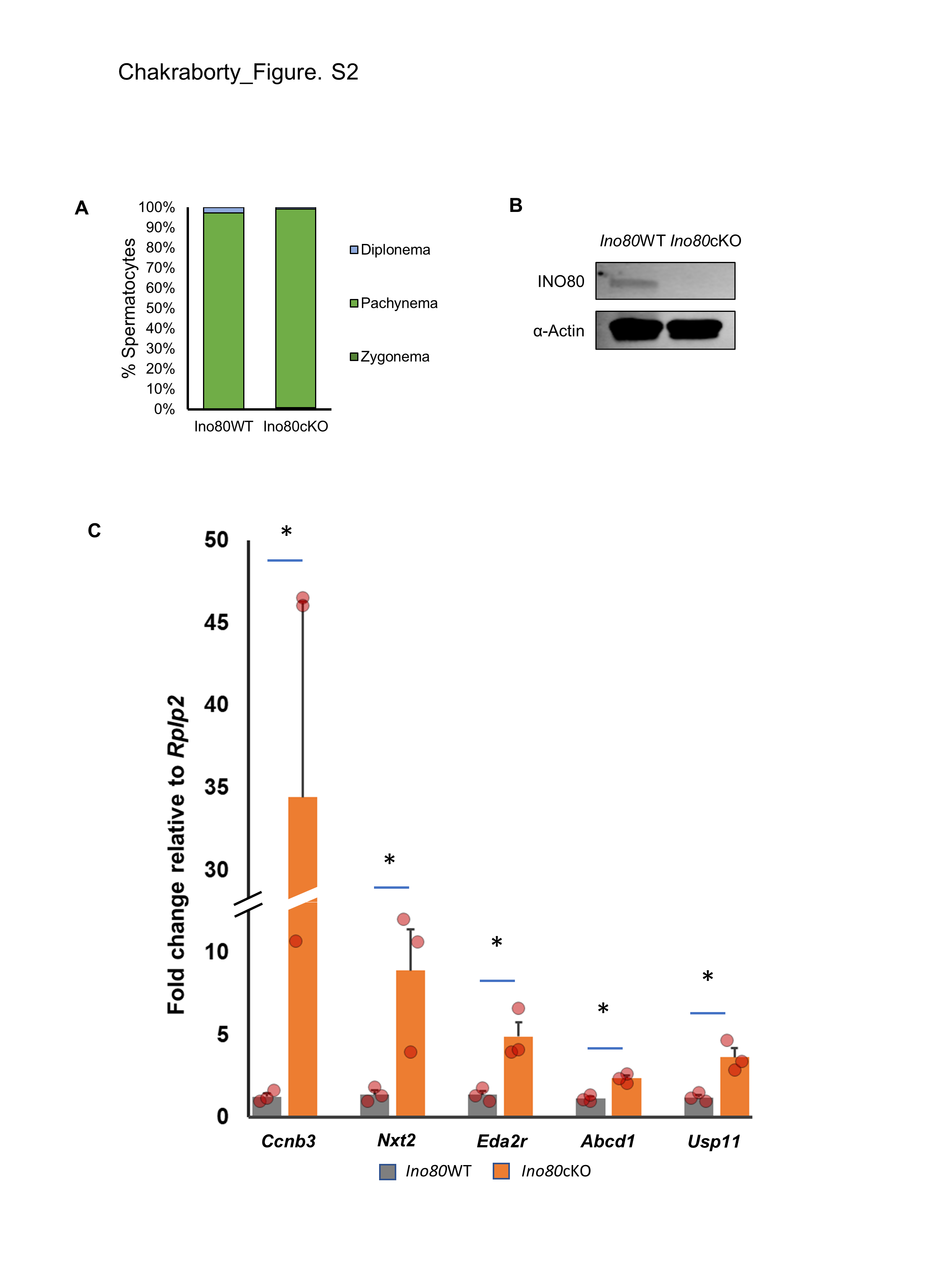

Supplement: S2 Fig — (A) Comparison of spermatocyte population in synchronized P24 Ino80WT and Ino80cKO testes. (B) Immunoblot for INO80 and alpha-actin in synchronized P24 Ino80WT and Ino80cKO testes. (C) Quantitative RT-PCR analysis of representative sex-linked gene expression levels normalized to Rplp2 in synchronized P24 Ino80WT and Ino80cKO testes. Bars represent mean ± s.e.m. *; p<0.05, as calculated by unpaired t-test (n = 3) (TIF) [file pgen.1011431.s002.TIF]

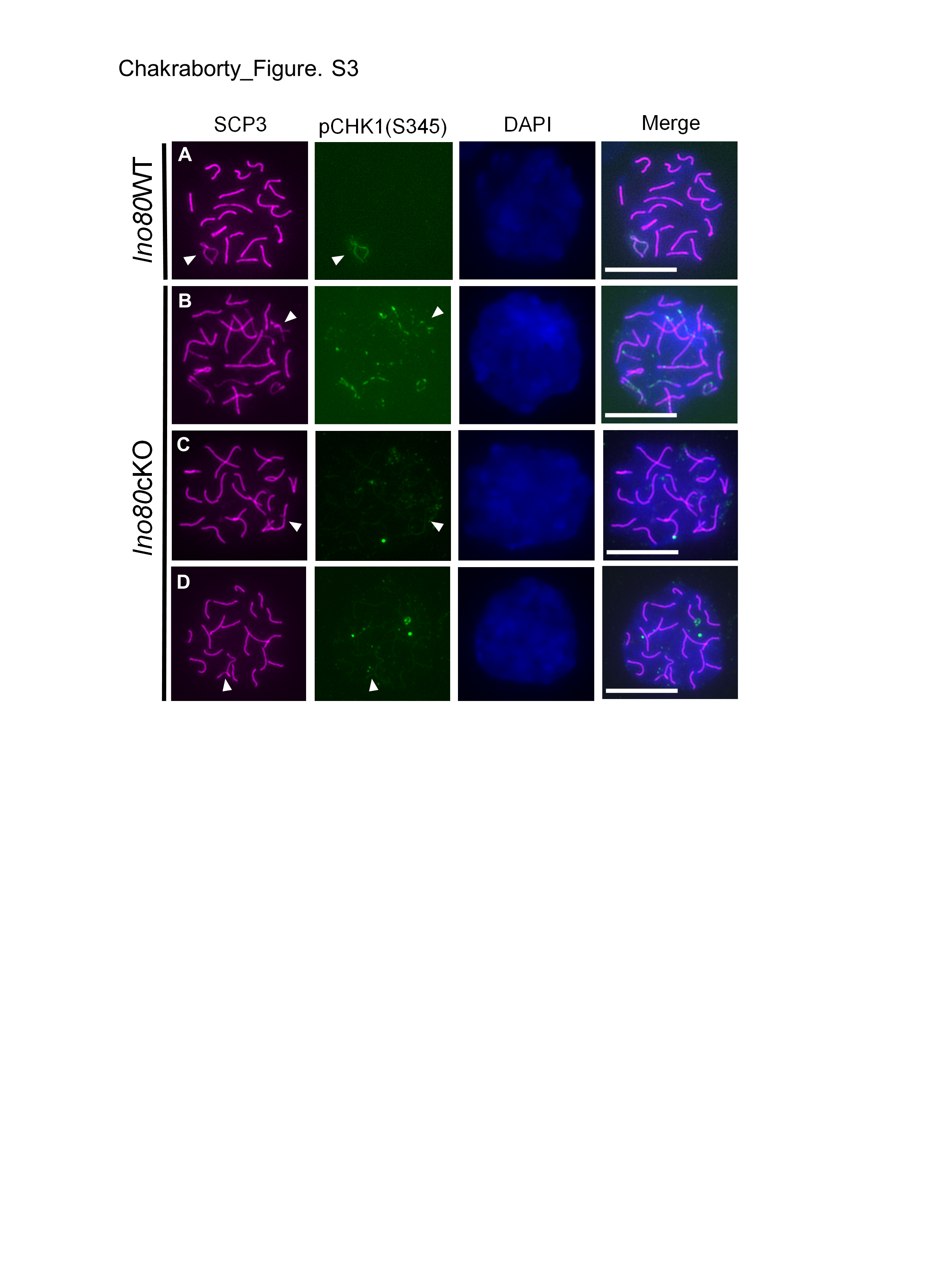

Supplement: S3 Fig — (A-D) Immunolocalization of SCP3 (magenta) and pCHK1(S345) (green) in Ino80WT (A) or Ino80cKO (B-D) spermatocytes. Autosomes demonstrate pCHK1(S345) signal at sites with incomplete synapsis (B), while sex chromosomes at pachynema exhibit aberrant pCHK1 level in Ino80cKO spermatocytes (C-D). DAPI is shown in blue. Scale bar = 10μM. White arrowhead; sex-chromosome. (TIF) [file pgen.1011431.s003.TIF]

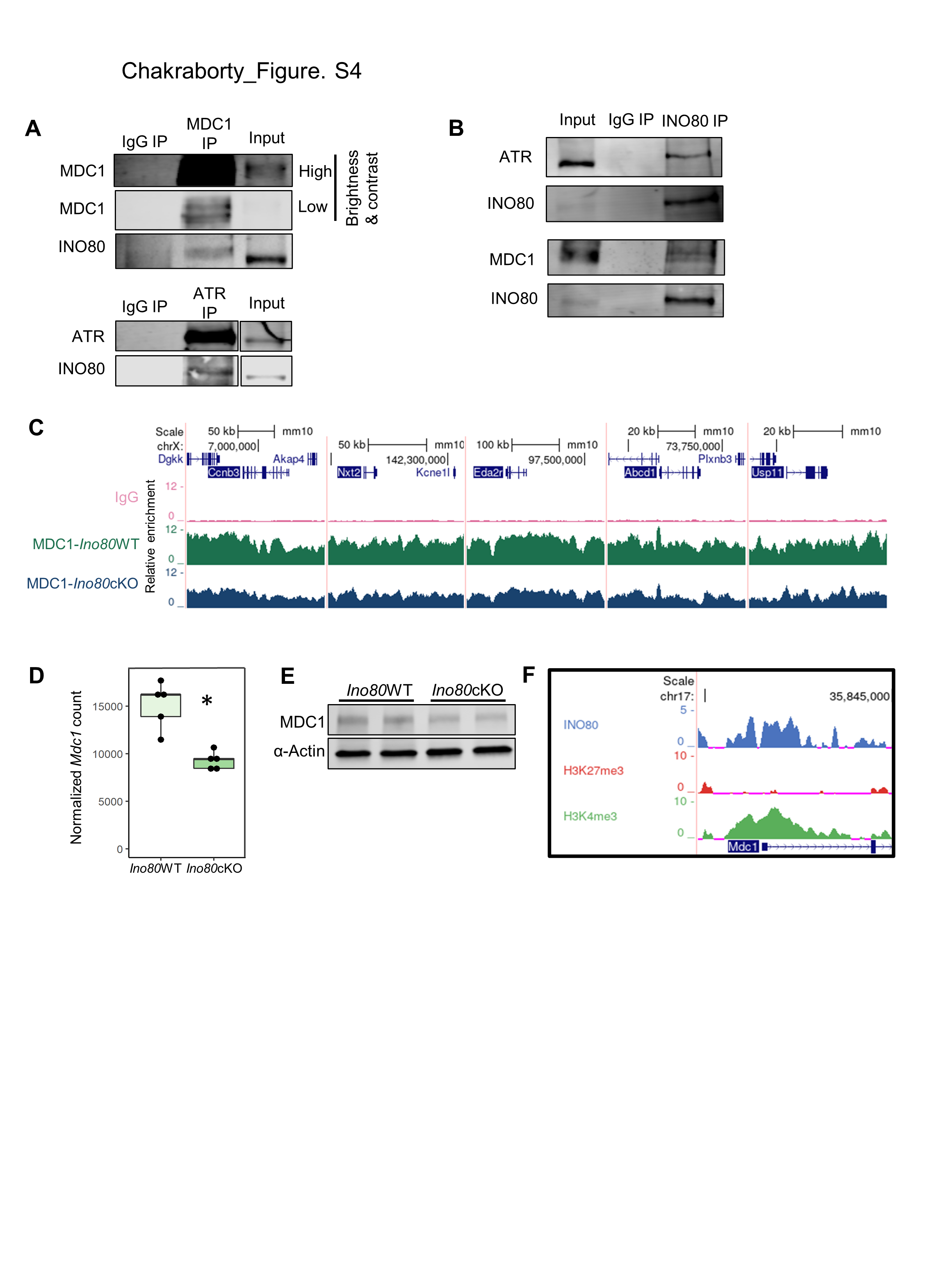

Supplement: S4 Fig — (A) Immunoblot images demonstrate the interaction between INO80 with ATR and MDC1 by the presence of INO80 in both ATR and MDC1 immunoprecipitated samples. The top panel shows two brightness and contrast levels from the same blot to visualize MDC1 in input and immunoprecipitation samples. Spliced sections in the bottom panel are part of the same blot. (B) Immunoblot images demonstrate the presence of ATR and MDC1 in INO80-immunoprecipitated sample in the presence of DNase I. (C) Genomic tracks depicting the normalized enrichment of MDC1 at the representative sex-linked DEGs in Ino80WT and Ino80cKO pachytene spermatocytes. (D) Boxplot showing the normalized count of Mdc1 transcripts from Ino80WT and Ino80cKO spermatocytes. (n = 5) (Analyzed from GEO Dataset GSE179584) [32] (E) Immunoblot showing MDC1 (top) and α-Actin (bottom) expression from Ino80WT and Ino80cKO spermatocytes on P18. (F) Genomic tracks illustrated the enrichment of INO80, H3K27me3, and H3K4me3 at the Mdc1 promoter-proximal area. (Analyzed from GEO Dataset GSE179584) [32] (TIF) [file pgen.1011431.s004.TIF]

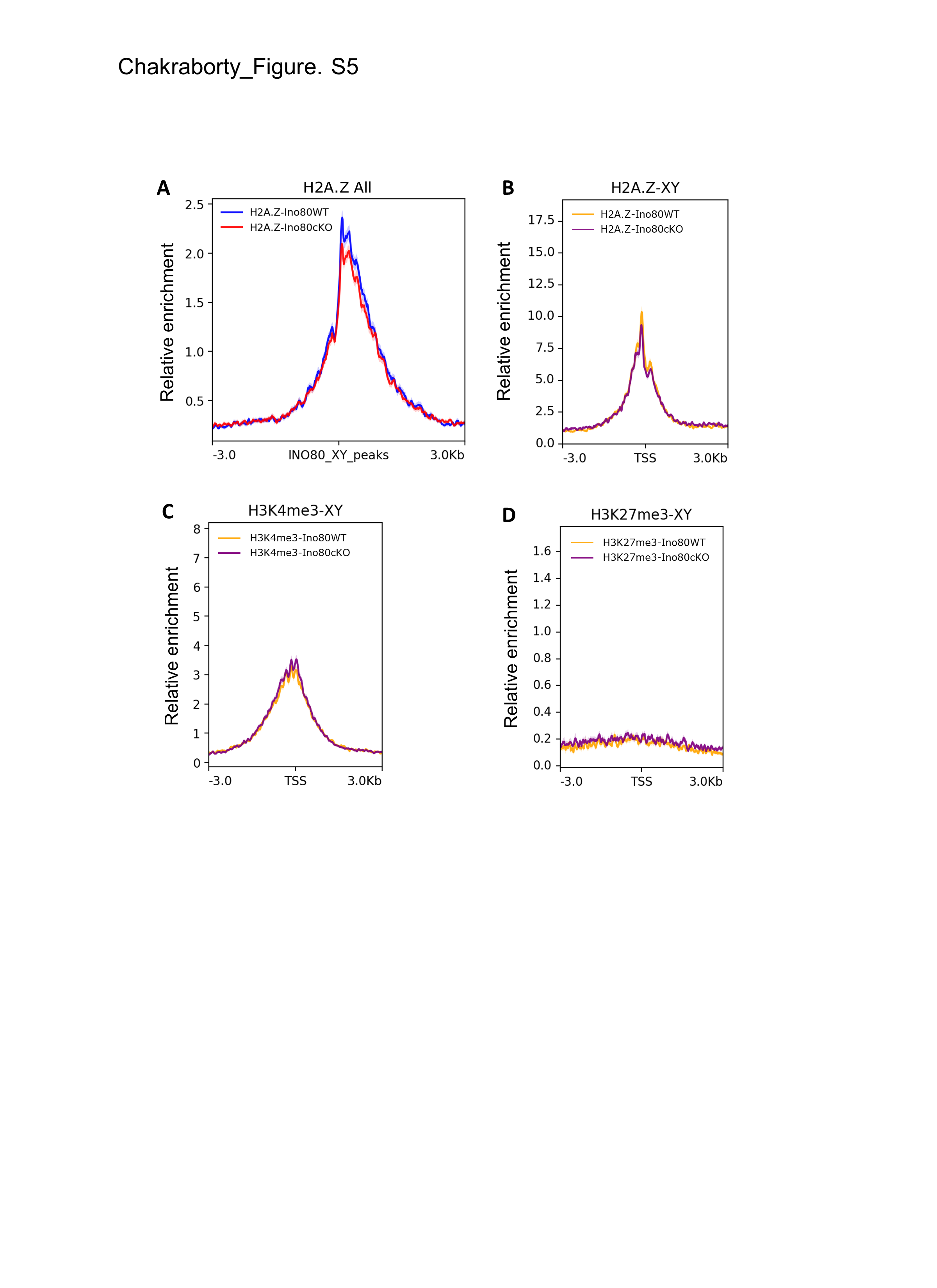

Supplement: S5 Fig — Metaplot illustrating changes in H2A.Z (A-B), H3K4me3 (C), and H3K27me3 (D) occupancy at either INO80 binding sites (A) or promoter/TSS regions (B-D) in sex chromosomes (Analyzed from GEO Dataset GSE179584) [32]. (TIF) [file pgen.1011431.s005.TIF]

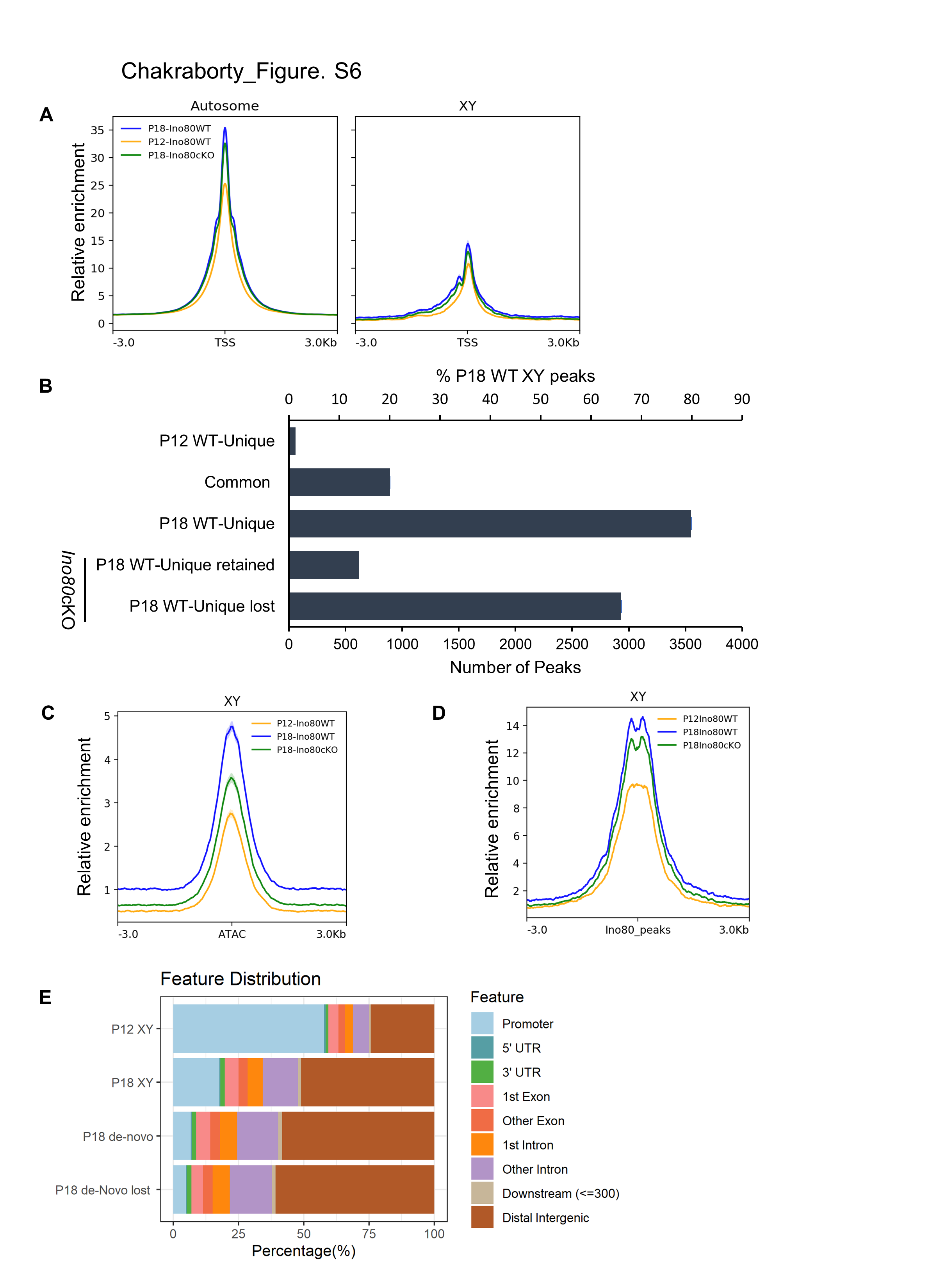

Supplement: S6 Fig — (A) Metaplot illustrating chromatin accessibility at the promoter/TSS regions of autosomes and sex chromosomes in either P12 Ino80WT or P18 Ino80WT and Ino80cKO spermatocytes. (B) Comparison of ATAC peaks at sex chromosomes during the transition from P12 spermatocyte to P18 spermatocyte and in P18 Ino80WT vs. Ino80cKO spermatocytes. (C-D) Metaplot illustrating chromatin accessibility at the sex chromosomes in P12 Ino80WT and P18 Ino80WT and Ino80cKO spermatocytes at all the ATAC peaks (C) and at the INO80 peaks (D). (E) Genomic annotation of ATAC-peaks in P12 and P18 spermatocytes, as well as the de novo peaks generated during pachynema transition during P18 and lost due to Ino80 deletion. P18 ATAC-seq data was analyzed from GEO Dataset GSE179584 [32]. (TIF) [file pgen.1011431.s006.TIF]

Chakraborty\_ Fig. 3H WB images

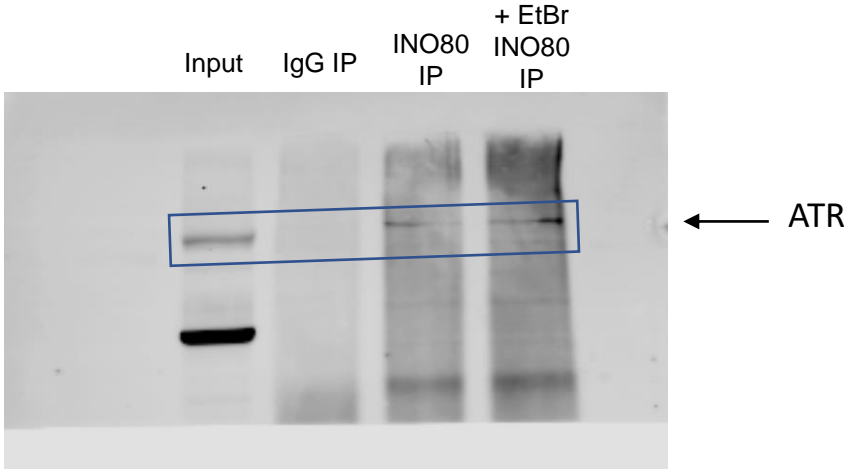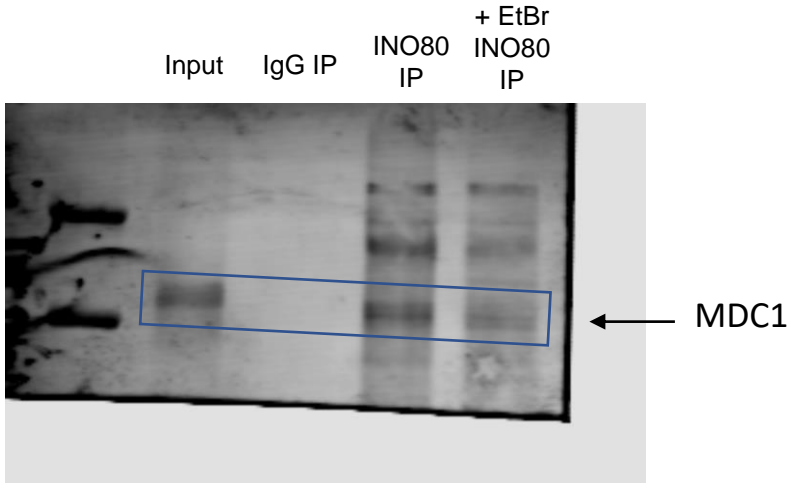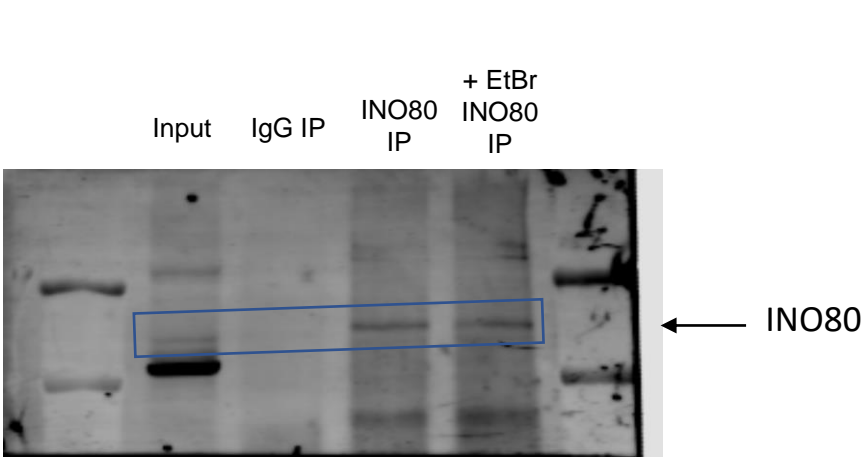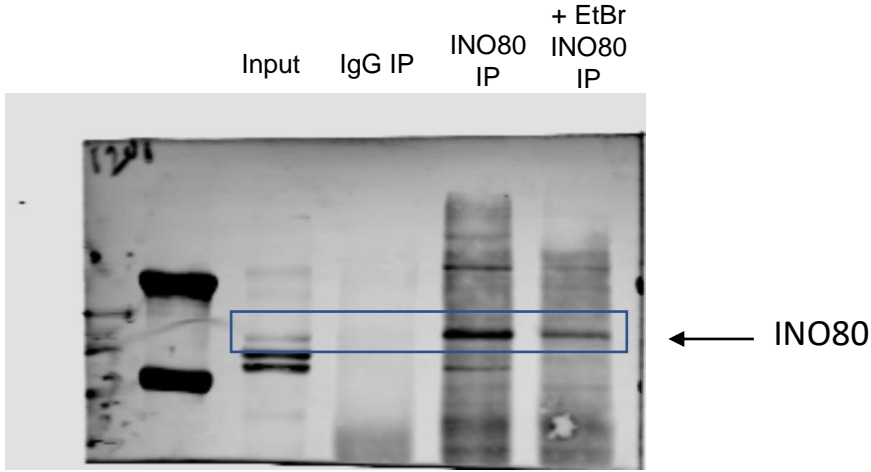

Chakraborty\_ Fig. S2B WB image

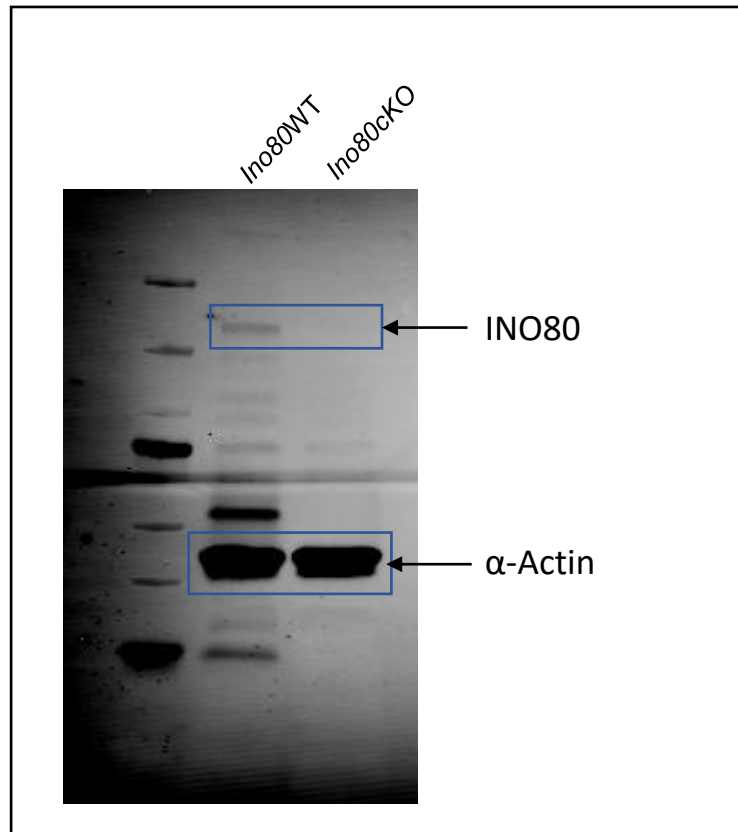

Chakraborty\_Fig. S4 WB images

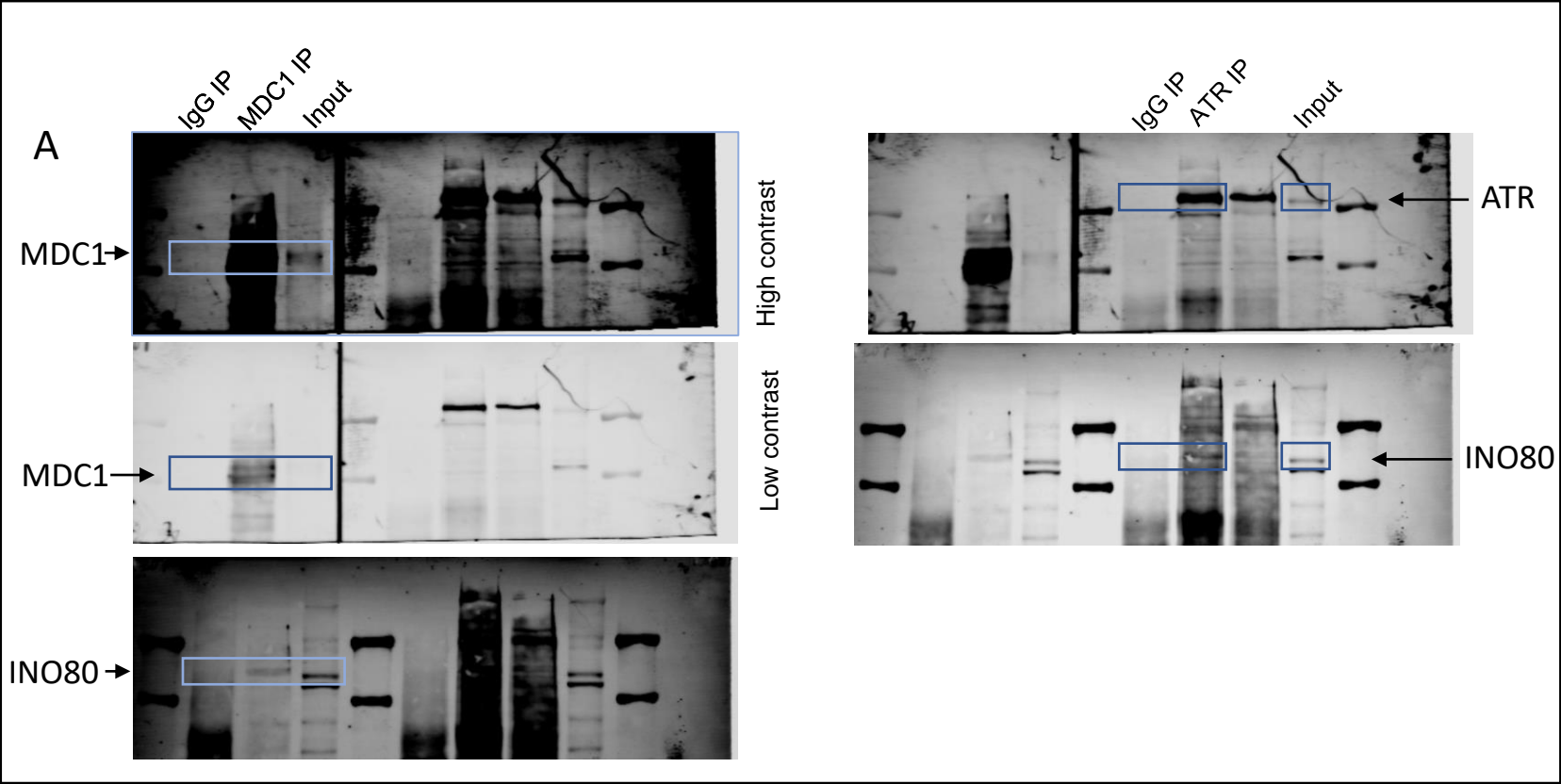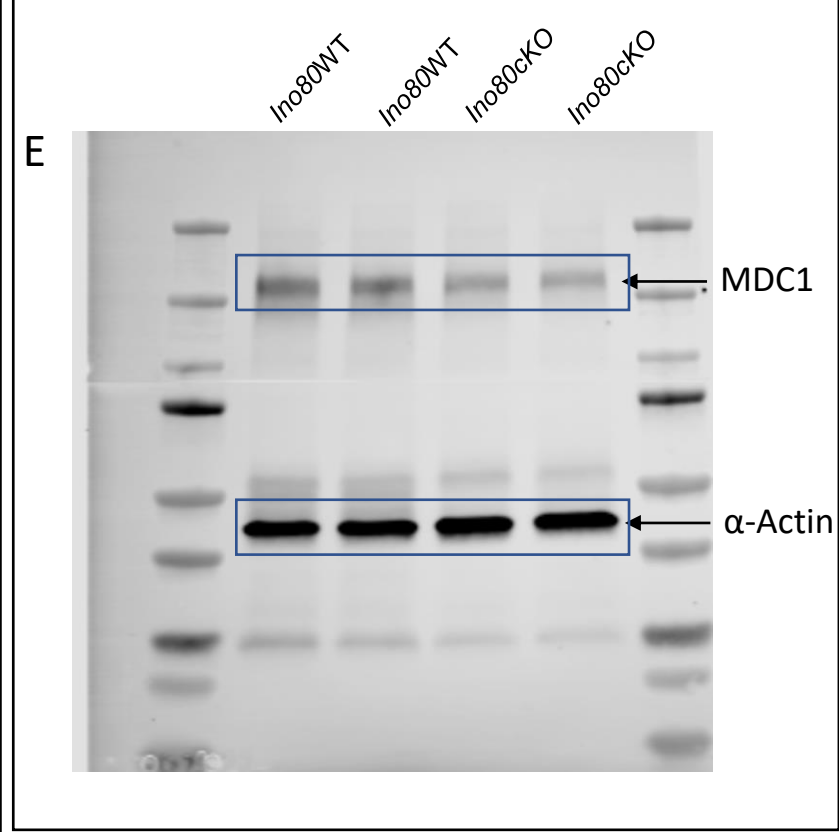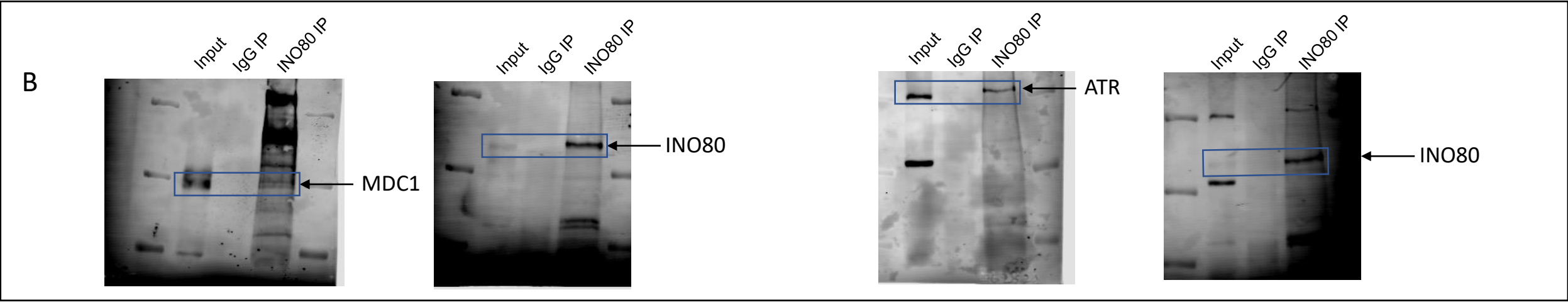

Supplement: S1 File — (PDF) [file pgen.1011431.s013.pdf]
