# Supplementary material for: INO80 regulates chromatin accessibility to facilitate suppression of sex-linked gene expression during mouse spermatogenesis
Source: PLoS Genet. 2024 Oct 15;20(10):e1011431. doi: 10.1371/journal.pgen.1011431 (PMC11508167; doi:10.1371/journal.pgen.1011431)
Supplement: S3 Table — (DOC) [file pgen.1011431.s009.doc]

Table S3: Genotyping primers used in this study.

| **Allele** | **Sequence** |
| --- | --- |
| Ino80 floxed forward | 5'-GATACTTCTGCCTCCACACTTC-3' |
| Ino80 floxed reverse | 5'-CTGGCACCTTTCCAGTCTTT-3' |
| Ino80 excised forward | 5'-TGTGTAGCAACCTACAGCTA-3' |
| Ino80 excised reverse | 5'-GTTGCTGTGTCTTTGCTTTG-3' |
| Stra8Cre forward | 5'-GTGCAAGCTGAACAACAGGA-3' |
| Stra8Cre reverse | 5'-AGGGACACAGCATTGGAGTC-3' |
