# Supplementary material for: INO80 regulates chromatin accessibility to facilitate suppression of sex-linked gene expression during mouse spermatogenesis
Source: PLoS Genet. 2024 Oct 15;20(10):e1011431. doi: 10.1371/journal.pgen.1011431 (PMC11508167; doi:10.1371/journal.pgen.1011431)
Supplement: S4 Table — (DOC) [file pgen.1011431.s010.doc]

Table S4: Quantitative PCR primers used in this study.

| **Region/Gene** | **Primer** |
| --- | --- |
| Ccnb3 Forward | 5'-GCTCACCTCAAGCCCATTAT-3' |
| Ccnb3 Reverse | 5'-TTGACTGGTGGCTTCTCTTTAG-3' |
| Nxt2 Forward | 5'-GTAGAGCTGCCGAGGAATTT-3' |
| Nxt2 Reverse | 5'-TCCAGATTAGAGTGGCTTTGTC-3' |
| Eda2r Forward | 5'-CCAGTTGAGCTTAGTGAAGGTAG-3' |
| Eda2r Reverse | 5'-AGGAAGGCCAGAGCAAATAC -3' |
| Abcd1 Forward | 5'-TGGATGGACGACTTCGAAAC-3' |
| Abcd1 Reverse | 5'-GGCTTGGTCAGGTTGGAATA-3' |
| Usp11 Forward | 5'-GCTCGTTCAGCACAGTGATA-3' |
| Usp11 Reverse | 5'-TCCTGAGGCTCTACCAGAAA-3' |
| Rplp2 Forward | 5'-CCTAGCGCCAAAGACATCAA-3' |
| Rplp2 Reverse | 5'-GACCTTGTTGAGCCGATCAT-3' |
