# Supplementary material for: INO80 regulates chromatin accessibility to facilitate suppression of sex-linked gene expression during mouse spermatogenesis
Source: PLoS Genet. 2024 Oct 15;20(10):e1011431. doi: 10.1371/journal.pgen.1011431 (PMC11508167; doi:10.1371/journal.pgen.1011431)
Supplement: S5 Table — (DOC) [file pgen.1011431.s011.doc]

Table S5: Primary antibodies used in this study.

| Primary antibody | Source | Amount/Dilution |
| --- | --- | --- |
| Rabbit anti-INO80 | Abcam (ab105451) | ChIP-seq (10μg)  WB (1:2000)  IF (1:700) |
| Rabbit anti-INO80 | Novus Biologicals (NBP1-78758) | IP (10 μg)  IF (1:500) |
| Rabbit anti-ATR | Abcam (ab2905) | IP (10 μg) |
| Rabbit anti-ATR | Millipore (09-070) | WB 1:1000 |
| Goat anti-ATR | Santa Cruz (sc-1887) | IF (1:50) |
| Rabbit anti phospho-CHK1(Ser345) | Cell signaling (2348) | IF (1:200) |
| Mouse anti-MDC1 | Novus Biologicals (NBP2-12890) | WB (1:1000)  IF (1:200)  CUT&RUN (1:200) |
| Mouse anti-alpha-Actin | Santa Cruz (sc-32251) | WB 1:2000 |
| Mouse anti-SYCP3 | Abcam (ab97672) | IF 1:1000 |
| Rabbit anti-SYCP3 | Novus Biologicals (NB300-231) | IF (1:200) |
| Goat anti phosphor RNA Polymerase II (Ser2) | Active Motif (61084) | IF: (1:200) |
| Mouse anti-γH2A.X | Millipore (05-636) | IF (1:1500) |
