# Supplementary material for: INO80 regulates chromatin accessibility to facilitate suppression of sex-linked gene expression during mouse spermatogenesis
Source: PLoS Genet. 2024 Oct 15;20(10):e1011431. doi: 10.1371/journal.pgen.1011431 (PMC11508167; doi:10.1371/journal.pgen.1011431)
Supplement: Attachment — Submitted filename: ResponsetoReviews.docx [file pgen.1011431.s014.docx]

Reviewer #1:

1. Notably, INO80 is not a protein specifically expressed during meiosis, and I am curious why it plays a specific role at this developmental stage, a question that seems unanswered in this paper.

Response: Most chromatin remodelers, including INO80, are expressed in several tissues and play an essential role in several developmental and disease pathways. INO80's cell type or developmental stage-specific roles are dictated by the availability and actions of other stage and cell type-specific proteins (co-factors) responsible for specific functions. INO80 regulates gene expression or genomic interaction of regulatory factors through chromatin remodeling. We have added lines 78-87 in the introduction section to address this.

2. Additionally, spermatocytes lacking INO80 exhibit conjoint abnormalities, with many analyzed cells possibly not in the pachytene but in an abnormal leptotene stage. This raises concerns that some conclusions about MSCI anomalies may be due to incomplete development rather than specific involvement of INO80.

Response: The analyzed cells displayed synapsed X-chromosomes at the pseudo-autosomal region (PAR) and the characteristic structure of the sex chromosomes during pachynema. Therefore, it is unlikely that the cells were in leptonema. Based on the overall gene expression signature, *Ino80* mutant testes had no change in zygonema at P18 (1). We observed the reduced suppression of sex-linked gene expression in *Ino80* mutant spermatocytes, where INO80 interacts with proteins such as MDC1, which is responsible for MSCI. Therefore, it is logical to conclude that INO80 plays a role in establishing sex-linked gene suppression during early pachynema.

3. In Figures 1 A and B, the fluorescent localization of INO80 seems to have been presented in the paper Development (2023) 150, dev202158 and does not constitute new evidence. What does the Y-axis represent in Figure C? The conditions of other chromosomes could also be presented to more clearly delineate the distribution of INO80 across different chromosomes.

Response: While we have presented a low magnification image of INO80 immunolocalization in the *Ino80*WT and *Ino80*cKO testes in the previous report mentioned (1), additional images were necessary to appreciate the immunolocalization of INO80 at the sex chromosomes of pachytene spermatocytes. Therefore, we included previously unpublished IF images of spermatocyte spreads at higher magnification for clarity and a new image of the seminiferous tubule harboring several pachytene spermatocytes to suggest its distribution. These INO80 immunolocalization images in Figure 1 demonstrate the localization of INO80 at the sex chromosomes.

The Y-axis in Fig. 1C represents the relative enrichment of INO80. We apologize for this oversight and modified the figure to add the Y-axis legend. We have added an additional metaplot in Fig.1C, displaying INO80 occupancy at the autosomes.

4. Although ATR is shown to decrease in the marked area in Figure 3 G, it is noteworthy that other synaptonemal complex regions appear to be upregulated. Moreover, judging from SYCP3, it seems this cell has additional synapsis issues. Please consider whether including such cells in the analysis of pachytene spermatocytes could impact the results. A similar situation exists in Fig.S3.

Response: We have analyzed cells that show synapsed sex chromosomes and autosomes, and we have included the representative image of abnormal *Ino80* mutant spermatocytes to show the aberrant distribution of ATR along the X-Y axis. The presence of ATR at the autosomes is indicative of incomplete DNA damage repair. However, its normal distribution at the synapsed sex chromosomes was also affected. As INO80 targets and regulates accessibility of the DNA damage repair sites at the sex chromosomes and interacts with and affects ATR distribution at the sex chromosomes, we conclude that INO80 is involved in the localization of ATR.

5. For Figures 4 and S4, it would be better to use cKO as a control instead of IgG.

Response: We have demonstrated the specificity of INO80 interaction with ATR and MDC1 by forward and reverse immunoprecipitation. We have detected ATR and MDC1 upon immunoprecipitation of INO80, as well as detected INO80 upon reverse immunoprecipitation of either ATR or MDC1. INO80 IP was performed by accumulating the spermatocytes isolated from 10-15 age-matched prepubertal WT mice. However, based on the low frequency of the generation of conditional *Ino80* mutant mice, it is not feasible to use *Ino80* mutant testis for immunoprecipitation due to the requirement for a large number of mutant spermatocytes for each experiment.

6. Figure 7 (now Figure 8) could be revised to reflect the structural model of the Hexasome-INO80 complex revealed in Science 381, 313–319 (2023), to update the depiction of the INO80 action model.

Response: We have updated the model to depict the INO80-nucleosome interaction. However, unsure of the hexasome-INO80 interaction in the meiotic sex chromosomes, we refrained from indicating the presence of the hexasome in the model. Instead, we have added this possible mechanism in the discussion section on lines 330-338.

Reviewer #2:

1. Fig S1: can the authors provide metagene plots to show whether gamma-H2AX co-localizes with INO80, both on autosomes vs. sex chromosomes?

Response: We have added a metagene plot in Fig. S1C to show the co-localization of INO80 and γH2A.X at the sex chromosomes. However, in the analyzed γH2A.X ChIP-seq dataset, γH2A.X occupancy during pachynema is absent from autosomes, and therefore, co-localization could not be determined at autosomes.

2. Fig 4H: can the authors carry out INO80 IP in the presence of DNase?

Response: We have performed INO80 IP in the presence of DNase. The results are presented in Fig. S4B and explained in the results section (lines 169-171). In brief, we have observed the interactions between INO80 and either MDC1 or ATR in the presence of DNase.

3. Fig S4A: can the authors sequence the MDC1 Cut&Run samples to better examine MDC1 occupancy in WT vs. Ino80 KO spermatocytes?

Response: We have performed MDC1 CUT&RUN sequencing, and the results are presented in the new Fig. 5, along with modified text (lines 174-186) in the results section. In brief, we observed robust MDC1 occupancy at the INO80-binding sites in the WT sex chromosomes and a reduction of MDC1 binding in the *Ino80* mutant sex chromosomes.

4. Fig 5 (now Fig 6): similarly, can the authors carry out gammH2AX Cut&Run-seq to examine its occupancy in WT vs. Ino80 KO spermatocytes? Together with the above, these genomic experiments are necessary to strengthen the conclusion that INO80 is required for DSBR factor recruitment.

Response: To perform CUT&RUN, we have tested two different antibodies, both of which show specific staining in IF experiments for γH2A.X in murine spermatocytes. However, we could not achieve suitable enrichment of γH2A.X after performing CUT&RUN for γH2A.X multiple times, and therefore, we consider the experiment not feasible at this time.

5. Fig S6A: since most of gained ATAC-peaks from P12 to P18 are not at TSS, can the authors provide metagene plots to show ATAC-seq signal centered on ATAC-seq peak center (instead of TSS) in P12 WT, P18 WT and P18 Ino80 KO? This will show whether INO80 is required for the enhanced chromatin accessibility at these non-TSS sites.

Response: We have added a metagene plot (Fig. S6C) showing the ATAC-seq signal centered at ATAC peaks. The letter identifiers of the following panels were changed accordingly. We observed a moderate reduction in the accessibility among all ATAC-seq peaks.

6. Fig 6C (now Fig 7C): to better connect the changes in ATAC-seq signal with changes in sex chromosome gene expression after Ino80 deletion, can the authors examine whether de-repressed sex chromosome genes in Fig 2A-B are over-represented in the nearest genes with differential ATAC-seq peaks in Fig 6C?

Response: We performed gene set enrichment analysis with the RNAseq data to determine enrichment with the genes near the reduced accessible regions at sex chromosomes in *Ino80* mutant spermatocytes. We observed significant enrichment of the nearest sex-linked genes from the ATAC-seq peaks, which is included in Fig. 7E. We have also described this result in lines 261-264 (lines 260-263).

7. Fig 6E (Now Fig 7F): can the authors provide box plot or metagene plot to show changes in ATAC-seq signal at INO80-bound regions between WT vs. Ino80 KO?

Response: We have added a metagene plot showing the ATAC-seq signal at INO80-peaks between Ino80WT and cKO (Fig. S6D), described in the results section in lines 229-232.

Reviewer #3:

1. The introduction is hard to follow. It would help if the authors explained how MSCI and the sex body are related. Paragraphs 2 and 3 could be consolidated and untangled to describe the temporal order of events for clarity.

Response: We altered the text as suggested.

2. INO80 is not mentioned until line 82. It would be helpful to describe this factor and explain why it drew your attention in this study.

Response: We have added more information about the expression and activity of INO80 in the introduction section on lines 78-87.

3. It would also be helpful to start a new paragraph at line 66 “MSCI is initiated…” as it is very important throughout the rest of the paper that the audience remembers that DSBs initiate MSCI.

Response: We started a new paragraph as suggested.

4. The last paragraph is repetitive, and confusing. The statement that INO80 “promotes the opening of sex chromatin during the zygonema-to-pachylema transition” (here and in the abstract) is conceptually difficult when the paragraph is about silencing of sex-linked genes. The authors need to find a way to unpack this sentence.

Response: We have modified the wording to “Further, INO80 promotes the opening of the sex chromatin during the zygonema-to-pachynema transition at the DSB regions. INO80 also facilitates the recruitment of DNA repair factors at the DSB sites and interacts with DSBR factors to facilitate the silencing of sex chromosomes.”

5. Results - Fig. 1
   - A main finding of this manuscript is that INO80 is enriched at the sex chromosomes (and sex body), in mid/late pachynema. The authors show this with ChIP-seq data but INO80 localization in meiotic spreads throughout prophase I (leptonema, zygonema, pachynema) should demonstrate that INO80 is not enriched at the sex chromosome in early prophase, but only in pachynema.

Response: We have presented INO80 ChIP-seq data in Fig. 1C, demonstrating that INO80 is localized at the sex chromosomes at both zygonema and pachynema and is more profound during pachynema. It is possible that chromatin remodeler INO80 may be important for other cellular events during stages preceding pachynema by enabling other regulatory proteins to localize at their target genomic sites and perform separate functions.

6. - Co-staining with other markers of the sex body would validate the findings, and could show which proteins of the sex body become enriched in a similar pattern or with similar timing.

Response: We have observed aberrant localization of the markers of the sex body, such as H2A.X and MDC1, which prompted us to use the structure of the partially synapsed sex chromosomes to determine the pachynema stage.

7. - At the end of figure 1 results, line 100, “These data suggested a possible role of INO80 in DSB repair…” but, I believe the authors actually mean “a role in MSCI initiation”. This is an important distinction.

Response: Based on its robust localization in Fig. 1, we speculated that INO80 may be important in DSB repair factors' localization at the sex chromosomes and suppression of sex-linked genes because these two major events occur at the sex chromosomes during pachynema. We have changed it to “DSB repair factor localization and silencing the expression of genes on the sex chromosomes.”

8. Line 129 - 131: Is there a sex body in the absence of INO80?

Response: The aberrant localization of H2A.X and MDC1 indicates that the sex body formation is disrupted in *Ino80* mutant spermatocytes.

9. Figure 2 – this figure needs an IF marker for the sex body to show that POL II is not excluded. Is the problem that all sex body markers are gone in the absence of INO80? If this is the case, the authors need to explain that POL II is present in the region of the XY chromosome pair. I don’t think you can say it is absent from the “sex body” if you can’t label it.

Response: We have changed it to sex chromosomes.

10. Figure 3 – In this figure, images in A,C, and G are dim which raises questions about whether the image is good enough to see ATR if it were present. The nuclei in A and G are very small. Is this significant? Or is the mag different here?

Response: The images for ATR (green) were captured using the same settings for all, including magnification. As ATR staining at the sex chromosomes is visible in the WT cells, we expect it to be visible in the mutant cells as well, where it is present.

11. Figure 4F – This figure has a similar problem. The SYCP3 label is dim which raises questions about whether the level of MDC1 is actually lower than wild type. Please replace these images with a different sample.

Response: SYCP3 and MDC1 signals were captured at separate fluorescent channels with channel-specific and mutually exclusive exposure settings, which remain constant from sample to sample. So, a change in SYCP3 intensity is not expected to cause any similar change in the MDC1 signal level.

12. Figure 5 (Now Figure 6) – Most of the text suggests that the authors believe that INO80 amplifies gH2AX in loops, but in some mutant samples, I don’t see any gH2AX. Do the authors believe that DSBs happen in the absence of INO80? Do DSBs occur at consistent sites? Does Spo11 make these breaks? A little more information about this literature would be helpful. I also found it strange that DNA is not required for interaction of INO80 with MDC1 and ATR. I was thinking that INO80 was attracted to DSBs and recruited the other components of the complex. Other readers likely also need further explanation of this section.

Response: Meiotic DSBs are believed to be non-random, occur at hotspots and eventually marked by γH2A.X (2), which we have included in introduction section at lines 53-54. We believe that SPO11-mediated DSB formation occurs normally in the *Ino80* mutants tested, as evident by γH2A.X localization in zygonema (3). We have mentioned this in lines 303-304. Regarding the interaction of INO80 with DSB repair proteins such as MDC1 and ATR, it is possible that INO80 is recruited to the DSB regions, and its interaction with ATR and MDC1 is necessary for the initial binding of these proteins at their target chromatin sites at the sex chromosomes. These explanations were included in the discussion section at lines 304-307.

13. Figure 6 (now Figure 7) -- The hypothesis that INO80 is needed for opening chromatin at DSBs to recruit other proteins involved in MSCI is very interesting. However, since it is counterintuitive that it is necessary to open chromatin to silence genes, this needs more careful explanation.

Response: We believe any DNA-binding protein-mediated activation or silencing of genes will need accessible chromatin for the proteins to access their target sites. Here, it is unclear how MDC1 recruitment silences the sex-linked genes, but the availability of open chromatin will facilitate MDC1 binding at DSB sites. We have revised the discussion section with this explanation on lines 329-338.

14. Line 477: manuscript should be lower case.

Response: We have corrected the typo.

15. Figure 2D: There is a circle in the first panel (SCP3 staining) that shouldn’t be there.

Response: We have removed the circle.

**References:**

1.	Chakraborty P, Magnuson T. INO80 requires a polycomb subunit to regulate the establishment of poised chromatin in murine spermatocytes. Development. 2022 Jan 1;149(1):1–12.

2.	Lange J, Yamada S, Tischfield SE, Pan J, Kim S, Zhu X, et al. The Landscape of Mouse Meiotic Double-Strand Break Formation, Processing, and Repair. Cell. 2016 Oct;167(3):695-708.e16.

3.	Serber DW, Runge JS, Menon DU, Magnuson T. The mouse INO80 chromatin-remodeling complex is an essential meiotic factor for spermatogenesis. Biol Reprod. 2016;94(1):1–9.
