# Supplementary material for: INO80 regulates chromatin accessibility to facilitate suppression of sex-linked gene expression during mouse spermatogenesis
Source: PLoS Genet. 2024 Oct 15;20(10):e1011431. doi: 10.1371/journal.pgen.1011431 (PMC11508167; doi:10.1371/journal.pgen.1011431)
Supplement: Attachment — Submitted filename: ResponsetoReviews.docx [file pgen.1011431.s015.docx]

Reviewer #1: The authors have made some revisions based on the reviewer's suggestions, but the following issues still persist:

1. Regarding the stage at which INO80 cKO spermatocytes are arrested, I find the authors' insistence on labeling these cells as pachytene stage to be highly debatable. Despite not providing a detailed classification, the images in Fig. S3 show that INO80 cKO spermatocytes exhibit incomplete synapsis in the SYCP3 signal, which is characteristic of zygotene arrest rather than pachytene. This incomplete assembly of the central element of the synaptonemal complex undermines the foundation of many of the authors' conclusive statements.

Response: Fig. S3 demonstrates that the ATR substrate CHK1 phosphorylation remains intact in *Ino80*cKO spermatocytes. pCHK1 in WT pachytene spermatocytes regresses to the sex chromosomes. However, pCHK1 is not present on the sex chromosomes of *Ino80*cKO pachytene spermatocytes (see new panels in Fig. S3c,d). Therefore, to demonstrate the presence of pCHK1 in the mutants, we chose an image with some incomplete autosomal synapses, which is rare. Further, the frequency of zygotene spermatocytes and the average gene expression levels from signature gene sets for zygonema do not change in *Ino80*cKO versus wild-type spermatocytes (Chakraborty and Magnuson, Development, 2022, PMID 35006254).

2. In Fig. 1C, the comparison of enrichment between the sex chromosomes and autosomes is not compelling. The sex chromosomes do not appear significantly enriched compared to autosomes overall, and some individual autosomes may even show higher enrichment than the sex chromosomes. However, the authors did not provide specific results for this.

Response: In Fig. 1C, we highlight the substantial localization of INO80 on the sex chromosomes at pachynema. In addition to having significant occupancy on the autosomes, there was an increased occupancy of INO80 on the sex chromosomes at P18 compared to zygonema. To clarify this point, we have modified the text in lines 113-114.

3. The role of INO80 in recruiting MDC1 and ATR lacks strong support from high-quality IP data. Additionally, there is an apparent absence of robust co-localization data.

Response: We have performed forward and reverse immunoprecipitation experiments to exhibit the interaction of INO80 with MDC1 and ATR. In addition to immunofluorescence localization of INO80 and MDC1 at the sex chromosomes, we have also shown occupancy of MDC1 at the INO80 binding sites at sex chromosomes by CUT&RUN, which suggests collaboration of INO80 and MDC1 at the sex chromosomes.

Reviewer #2: The authors have carried out additional experiments and analysis to address questions and comments from the previous review.

Response: We appreciate the recommendations from the reviewer.

Reviewer #3: This is an interesting story and the authors have done a good job of addressing my concerns in the body of the manuscript.
However, the presentation of figures could be improved. The in-figure labeling is minimal and often it is very difficult if not impossible to read.

Response: We thank the reviewer for the encouraging comment. We have increased the sizes of the in-figure labeling in Fig. 2B and Fig. 5A.
